# The lived experience of divorce: a narrative analysis of personal stories and identity reconstruction of women

**DOI:** 10.3389/fsoc.2025.1617489

**Published:** 2025-08-12

**Authors:** Ashalatha T. L., T. S. Saranya, Sandeep Kumar Gupta

**Affiliations:** Department of Psychology, CMR University, Bangalore, India

**Keywords:** divorce, identity reconstruction, lived experience, narrative analysis, psychological adjustment

## Abstract

This research investigates the lived perceptions of South Asian women in coping with the consequences of divorce, a culturally tainted break-up commonly fraught with shame, disposability, and loss of identity. Using narrative approach, from interviews and handpicked personal stories, the research investigates how women interpret post-divorce life in patriarchal, collectivist societies that value marriage as a pillar of feminine moral character. Based on Arthur Frank’s typology of illness stories, specifically the quest narrative, the results indicate that most women reinterpret their trauma as a transformative, resistant, and re-claimed journey. This research, however, explores an expansion of Frank’s model by incorporating the idea of the “agency quest,” where narrative coherence is supplemented by embodied and spiritual practices—journaling, yoga, chanting, and intuitive healing—as a part of identity reconstruction. Spirituality appeared not in the form of passive withdrawal but as an active ethical work by which women rethought the holy, recovered bodily sovereignty, and developed emotional toughness. The analysis locates these practices within paradigms of embodied cognition, feminist theology, and ethical self-cultivation, contending that healing is not just a cognitive or discursive endeavor but one profoundly embedded in sensory, affective, and ritual practice. Notably, the research considers the lack of chaos narratives and the structural limitations that dictate whose narratives get to be heard and told. It demands a feminist praxis affirming not just coherent narratives of development but the messiness, silence, and ambiguity that play a role in identity reconstruction following social disconnection.

## Introduction

Now an accepted global marital routine, divorce affects about 40 to 45% of first marriages, depending on jurisdictions (Amato, 2010). The crude divorce rate has remained quite low in India, with about 1 % of ever-married women being divorced or separated (Bose, 2024); nevertheless, the number of petitions filed in family courts has steadily risen in the last 20 years, indicating a gradual transformation in the patterns of marital stability and social attitude (Agnes, 1999; Parashar, 1992a,b). While the emotional and social consequences that follow divorce weigh heavily upon those most affected, especially women, such issues often take a backseat to their more systematic legal or economic consequences.

The psychosocial literature suggests that marital dissolution is not just a juridical event; rather, it is a biographical rupture that destabilises identity, social roles, and future projects (Bury, 1982; Kitson and Morgan, 1990). Long after the court decree is signed, women navigating this transition struggle with a variety of emotional issues, including grief, shame, anger, anxiety, and renegotiating social networks, finances, and caregiving responsibilities (Leopold, 2018). In much of South Asia, where a marriage is idealised as a lifelong sacrament and female respectability is tightly bound to marital status, these challenges become all the more intense (Derrett, 1978; Radhakrishnan, 2009).

### Narrative analysis as a lens

Qualitative narrative analysis gives us a window into these lived experiences by paying heed to the active construction of stories whereby individuals seek to interpret, negotiate, and communicate the meaning of disruptive events (Frank, 1995; Riessman, 2008). The language of divorce enables women to work through the loss, reclaim agency in the face of stigma, and express the identities they are constructing, which no longer include that of “wife.” Analysis of the structure, content, and cultural positioning of these narratives thus clarifies trajectories of coping and identity reconstruction, as well as the role of social support in post-divorce adjustment.

### Theoretical grounding

Arthur Frank’s (1995) typology of restitution, chaos, and quest narratives, originally developed for illness stories, has proved equally useful in understanding marital breakdown. Quest narratives, in which meaning is forged through adversity, often appear in accounts of women who frame divorce not as failure but as an opportunity for self-discovery and growth (Simon, 1995). These personal accounts unfold within gendered and cultural discourses that shape what can be said and how it can be said (Riessman, 2008).

### Sociocultural context of divorce in India

In the Indian setting, to speak of marital discord goes against norms that hold women’s endurance and family cohesion as ideals (Sharma A., 2004; Sharma U., 2004). According to Bose (2024), divorced women suffer a “double burden”: on one hand, coping with personal loss; on the other, societal stigma, economic insecurity, and pressure to remain silent. Therefore, narrative takes on an added significance: personal healing and a subtle defiance of cultural expectations. Analysis, then, of these storylines illuminates individual coping mechanisms while exposing the structural inequities under which women’s choices are made.

### Toward holistic, person-centred support

The global turn toward holistic, person-centred mental-health practice exemplified by trauma-informed and recovery-oriented approaches (Substance Abuse and Mental Health Services Administration, 2014) calls for integrating divorcing women’s subjective accounts into counselling, legal advocacy, and social-work interventions. Recognising narrative as an epistemic resource can foster empathetic, culturally sensitive services that move beyond fault-finding to address the emotional, social, and identity needs of women rebuilding their lives. By foregrounding women’s voices within their sociocultural milieu, the present study seeks to humanise divorce as a complex psychosocial phenomenon and to advocate for support systems that honour self-respect, mutual well-being, and the possibility of constructive life reconstruction.

Even as India’s crude divorce rate is statistically low, recent mixed-methods studies suggest a rising tide of divorce petitions among urban, middle-class women who cite emotional neglect, psychological abuse, or incompatibility. This increased visibility contradicts traditional beliefs about marital permanence, but it also places divorced women at risk of greater stigma and fewer institutional resources, particularly in collectivist settings where womanhood is equated with self-sacrifice and family solidarity. Researchers are increasingly claiming that while legal reforms have advanced, psychosocial support systems have not kept pace, particularly in supporting women with limited economic autonomy during their post-divorce transitions (Sarkar, 2024). This study addresses the critical gap by positioning women’s narratives as the source of healing, resistance, and rebuilding identity.

Concurrently, feminist scholars have started examining how South Asian women reconstruct selfhood on the moving terrain of class, urbanity, and global mobility. For example, Neupane (2024) illustrates the complex identity renegotiation that South Asian partners of international students undergo, many of whom face re-domestication and erasure in host nations, a liminality that resonates with the post-divorce transformations women go through as they transition in India. Recent empirical research also indicates that more educated women, better financially literate, and more easily accessible by urban areas are more inclined to construct “quest narratives” of post-divorce strength, while those with compounded vulnerabilities often get stuck within “chaos” narratives of abandonment and lowered self-esteem (Nigam, 2025; Lamb, 2022). This study stands to add to the growing corpus of intersectional feminist research by charting the narrative strategies that women use in reconstructing their roles, identities, and relationships after they experience marital breakdown (UN Women India, 2023).

Thus, new clinical research sustains the argument for reframing the narratives’ therapeutic potential for women who are divorced. Utilising a quasi-experimental design, Taheri Fard et al. (2023) found that an eight-week narrative therapy intervention reduced anxiety-generating thought processes in divorced single mothers. Correspondingly, found self-compassion and narrative coherence to have mediated divorce stigma and post-separation adjustment among women in India. These results validate the theoretical underpinnings of this study, which situates storytelling as not only a methodological necessity but also as an act of meaning-making that has the potential to reframe emotional pain into coherent identity work. By enabling women to rewrite their life narratives, narrative research is both an analytic instrument and a psychosocial intervention.

## Methodology

### Research design

This study adopts a qualitative methodological framework under narrative inquiry to understand the lived experiences of South Asian women affected by divorce and associated identity research. Narrative inquiry was chosen because of its focus on bringing to narrational orientation the richness of the dye of personal, culturally imbibed experiences, that is, intensely traumatic life transitions like divorce – allowing for deep interrogation of processes by which individuals make meaning and coherence following great ruptures of identity and stigma in their lives (Riessman, 2008). Narratives help the participants reconstruct identities and find ways of understanding these pathways that are relevant to divorce identity work and resilience (Clandinin and Connelly, 2000).

### Data sources

The study contains two major data sources:

#### Secondary data

The study’s secondary sources of data stem from three selected episodes of the Desi Divorces podcast, hosted by Dr. Ranjani Rao. Dr. Rao and her platform allow South Asian women to narrate their personal experiences of divorce in ways that emphasise emotional turmoil, the acquisition of resilience, and changes through the separation process. Dr. Rao, herself a divorcee and an author of the book Happily Ever After, provides an insider framework for interpreting the stories with understanding from personal and professional reading and lived experience. These episodes were chosen because they represented rich narrative diversity among the experiences being narrated. Transcriptions were made verbatim to preserve the emotional intensity and cultural nuances of the wordings so that the meaning-making process through these stories could be studied.

#### Primary data

In another instance, three individual in-depth interviews using semi-structured interviews with South Asian women experiencing divorce constituted the primary source of data. Participants were purposely sampled to cover variations in age, socio-economic status, and post-divorce experiences. Each interview lasted 60 to 90 min and was conducted over video call, with recording done following participants’ consent. The semi-structured design permitted participants’ spontaneous narration about their journey while allowing the interviewer to probe key issues relating to identity transformation and healing. All interviews were transcribed verbatim, keeping intact the affective tone and flow of narrative expressed by the participants.

### Data analysis

The data from both primary and secondary sources were analysed using thematic narrative analysis conducted following Riessman’s (2008) model. This consisted of a five-step process: (1) getting to know the data by multiple readings of transcripts, (2) preliminary open coding to isolate meaningful narrative portions, (3) aggregation of similar codes into overarching thematic categories, (4) building individual narratives within these themes to maintain the coherence and sequencing of participants’ stories, and (5) interpretive synthesis to uncover patterns, tensions, and culturally situated meanings across narratives. A five-stage analytic process was utilised to achieve methodological rigour and transparency:

#### Familiarisation with the data

We read each of the six transcripts (three interviews and three podcast episodes) repeatedly to get familiar with the tone, language, and emotional appeal of each story. Memo writing was undertaken during the initial analyses to record early impressions and contextual observations.

#### Initial (first-cycle) open coding

Via the NVivo 12 qualitative software, the transcripts were imported line by line for open coding. The method resulted in 142 early codes that captured a broad spectrum of emotional reactions, cultural tensions, coping strategies, identity disturbances, and narrative turning points. In-vivo codes (the words used by the participants) were kept to maintain authenticity.

#### Axial and thematic clustering

Associated codes were also aggregated into higher-order themes like identity disruption, coping mechanisms, relational processes, and narrative rewriting. These themes were further developed iteratively in discussions by the research team to match and ensure thematic saturation.

#### Narrative reconstruction and ordering

To maintain the temporal and narrative coherence of each case, individual narratives were reconstituted within the clustered themes. Care was taken regarding narrative structure, metaphors, plot developments, and silences towards Riessman’s insistence on content as well as structure.

#### Use of Frank’s typology (second-cycle coding)

Each was then coded under Frank’s (1995) narrative types: chaos (disorder, meaning loss), restitution (hope of return to normal), or quest (growth through adversity). One story (P4) took a chaos course; two (P2, P6) took restitution courses, and three (P1, P3, P5) depicted rich quest narratives with empowerment and spiritual reframing. This process further enhanced interpretive depth and facilitated cross-case comparison (see Table 1).

**Table 1:** Cross-case matrix.

Participant ID	Family support	Economic autonomy	Coping strategy	Narrative type	Identity reconstruction	Notes
P1(A)	Low	Moderate	Blogging, Online Forums	Quest	High	Reframed divorce as liberation
P2(B)	High	Low	Spiritual practices	Restitution	Moderate	Relied on faith and maternal role
P3(C)	Moderate	High	Psychotherapy	Quest	High	Focused on career reinvention
P4(X)	Minimal	Low	Avoidance, withdrawal	Chaos	Low	Expressed hopelessness, no active redefinition
P5(Y)	Moderate	High	Community activism	Quest	High	Became an advocate for divorced women
P6(Z)	Strong	Moderate	Journaling, peer support	Quest	Moderate-high	Balanced self-care with social work

To guarantee credibility and reliability, intercoder reliability was established through peer debriefing with an external qualitative researcher who coded two transcripts independently. Agreement on thematic categories was achieved at 85% consistency, and disagreements were negotiated through discussion. An audit trail was kept throughout the analytic process, including codebooks, memos, and version-controlled drafts.

This multi-layered method, coupling inductive coding, narrative structuring, and typological mapping, enabled both within-case depth and cross-case coherence, resulting in a rich understanding of how divorced South Asian women re-create meaning and identity through narrative.

### Ethical considerations

The study obtained ethical clearance from the Institutional Review Boards (IRB) of the university at which the study researcher is affiliated. The participants were fully informed regarding the objectives and procedures of the study and their right to withdraw from the study at any time without any consequence to them. Written consent was obtained from all participants. Pseudonyms were used in reporting the study, and all identifying information was removed from transcripts to ensure complete confidentiality. The study was thus conducted in adherence to ethical guidelines as outlined in the Declaration of Helsinki for studies involving human subjects.

## Results

For participants in this study, divorce was more than a legal or relational rupture; it was a profound disruption of identity, particularly for South Asian women who had been raised within traditional familial structures. Marriage had been intimately tied to cultural notions of respectability, womanhood, and self-worth. Divorce, therefore, marked not only the breaking of the relationship but also the dissolution of deeply internalised roles and long-held self-concepts. This identity disruption would leave many women with questions about who they were in the absence of a wife role.

A (38), brought up in a conservative family, expressed the intensity of this unravelling of the self:

“I wasn’t just ending a marriage; I was ending a version of myself that had existed since I was 21. I did not know who I was outside of that role.”

B (43), further elucidated the societal expectations-entwined experience as an awakening:

“Divorce wasn’t breaking me, it was revealing me. I had to peel back everything I had been told I was supposed to be.”

C (41), a marketing executive, highlighted the transformative element of rebuilding identity:

“I realised I was not broken. I was becoming. I had never seen myself so clearly.”

X (30), an assistant professor, echoed the sentiment of losing more than just a partner:

“I was not just fighting a failed marriage—I was grieving a version of my life I had worked so hard to build. I chose respect over suffering, and I hope that will be enough to carry me forward.”

Y (48), a mother of two, reflected on how her sense of self went down in flames after she discovered her husband’s affair:

“Inside, I was shattered. I kept thinking, was everything a lie? But yoga saved me. It made me feel like I had something to offer again.”

Z (47), after living through 16 years of emotional neglect and narcissistic abuse, viewed the divorce as an escape from illusion:

“I think I lived in an illusion all these years. When I could not take it anymore, I knew I had to save myself.”

Said transformation bears witness to Frank’s (1995) perspective of the quest narratives, wherein selfhood is reshaped through adversity. Under the passage of time, from disruption to reconstruction, the women turned away from externally imposed roles to identities authored by themselves, which were premised on autonomy, inner strength, and meaning (see Table 2).

**Table 2:** Identity shifts post-divorce.

Aspect of identity	Narrative disruption	Reconstruction process
Marital identity	Loss of social role as wife	Rebuilding autonomy through work, therapy
Self-perception	Shame, self-doubt, and guilt	Rediscovery of worth through self-love practices
Familial role	Shifting dynamics with parents and children	Reclaiming parental authority and emotional clarity
Purpose and agency	Feeling directionless, emotionally dependent	Development of purpose through healing and helping
Personal aspirations	Suppressed dreams and interests	Reconnection with creative and professional passions

### Coping mechanisms

Participants simultaneously adopt a layered and evolving coping mechanism to stem emotional upheaval. Included under these are cognitive reframing, therapeutic interventions, spiritual rituals, and creative expressions. The first instinct is usually maladaptive: isolation, denial—but the mature soul soon outgrows such measures. The changes in behaviour include moving from city A to city B, enrolling in new courses, exercising, or simply taking a holiday in Europe.

A found solace in solitude and nature.

*“I started taking morning walks again. Just me and the sunrise. That became my ritual. That was my recovery.”*

B turned to community healing circles and spiritual retreats:

*“I had to find places where I could speak my truth. Where I wasn’t ‘the divorced one,’ just a human being in healing.”*

C turned to poetry as a means of emotional processing.

*“I wrote every night. Not for anyone else. Just to hear myself think.”*

X: focused her energy on professional advancement:

*“I drowned myself in work and focused on becoming a better version of myself professionally.”*

Y healed by embodiment:

*“Yoga wasn’t just exercise – it was therapy. My body remembered how to trust before my mind did.”*

Z healed through expressive arts:

*“Some days I still feel lost, but dancing and singing – those are my lifelines now.”*

This transformation follows Lazarus and Folkman’s (1984) theory of coping, which describes coping as an evolving exchange between stressors and adaptive resources (see Table 3).

**Table 3:** Coping strategies across time.

Coping strategy type	Examples	Impact on recovery
Cognitive	Journaling, therapy, and self-help reading	Enhanced clarity, emotional processing
Behavioural	Daily routines, moving out, career shift	Regained control, built a new normal
Spiritual	Meditation, temple visits, chanting	Provided solace and existential grounding
Expressive	Writing, singing, painting	Released emotion, restored joy
Maladaptive (initial)	Isolation, denial, avoidance	Temporary numbing, prolonged grief

### Relational dynamics and social support

The nature of relational dynamics before, during, and after divorce deeply induced emotional consequences. For some, family became a pillar of strength; for others, a source of guilt and control. Thus, the reactions went from receiving empathy to exerting pressure in judgement. So these shady double acts equally made familial relationships both a heaven and a curse.

A daughter’s quiet words seemed to affirm this insight:

“Are you happier now, Amma? Because I like this version of you.”

B explained the importance of peer groups, the theatre of social media:

“Strangers in a support group held space for me in ways my family could not.”

C recalled the sting of patriarchal expectations:

“My father said, ‘A woman does not leave her marriage. She fixes it. That sentence rang in my head for months.”

X, in contrast, was bolstered by unconditional support:

“My parents never questioned me. That gave me strength. And my friends—they were my therapists.”

Y drew strength from her children:

*“My sons needed a strong mother. That was my reason to get up each day.”*

Z had little familial support but found comfort in her daughter’s company:

“It was friends and my daughter who showed up. I needed that – someone to just say, ‘You’re not alone.’

Support extended beyond families. Friends, colleagues, and online professionals harboured a safe environment for women to air their grievances and receive empathetic, non-judgemental support. These alternate supports helped many participants feel seen and emotionally anchored (see Table 4).

**Table 4:** Sources of support and relational impact.

Source of support	Perceived impact
Parents	Mixed—supportive in some, judgmental in others
Children	Often served as emotional anchors
Friends	Provided validation and active listening
Therapists	Guided emotional healing and perspective shift
Online Communities	Shared experiences, lessened isolation

### Meaning-making and rewriting the narrative

The divorce journey thus was one in meaning-making; journeys were narrative reconstruction. Divorce for the participants was transformed from being a symbol of brokenness into an act of courage and reclamation. With time, their positioning of divorce would transform from that of a failure to one of a threshold toward self-discovery and growth. This re-authoring gave participants the ability to reclaim their stories from dominant cultural scripts that had silenced them.

A: She described her journey as one of liberation:

“I finally gave myself permission to matter.”

B: Drew from nature metaphors:

“I’m like a river now—reshaping, flowing, but never stuck.”

C reframed her experience with clarity.

“I used to think divorce meant the end of love; now I know it can be the beginning of self-love.”

X spoke of reclaiming dignity:

“When I chose respect over suffering, I knew I was choosing myself.”

Y emphasised spiritual transformation:

“Yoga is not just what I do – it’s who I’ve become.”

Z shared a spiritual awakening:

“Even on the worst of days, I’ve started thanking the universe for giving me a second chance to love myself.”

These reflections characterise the concept of narrative re-authoring by White and Epston (1990), wherein the clients are allowed to reclaim themselves from dominant cultural narratives that no longer serve them. Spiritual and philosophical reflections were abundant. Several women drew on religious texts, nature metaphors, and personal rituals to symbolise the importance of their journey. Gratitude, too, became another theme, a witness to their pain and recognition of growth.

Table 5 presents a summary of major shifts in the narratives expressed by the participants through the different stages of their post-divorce processes, juxtaposing widely internalised cultural scripts surrounding marriage, womanhood, and social expectations with rewritten narratives through storytelling, healing, and the rebuilding of identity. The re-told stories involve much deeper meaning-making processes whereby these women positively contested and re-formed inherited stances that positioned divorce as failure, worthlessness, or isolation. Instead, their re-telling expressed an awakening, an assertion of self-worth, and resilience. This re-authoring of identity placed the participants squarely in their stories, away from societal taboos, as symbols of their integration and agency.

**Table 5:** Narrative themes and transformation.

Dominant cultural script	Rewritten narrative
“Divorce is failure”	“Divorce is an awakening.”
“Marriage defines a woman’s worth”	“My worth is self-defined.”
“Endure for the sake of family”	“Heal for the sake of wholeness.”
“You’re alone now”	“I am accompanied by myself.”
“It’s too late to start over”	“Every moment is a fresh beginning.”

For instance, the belief that “marriage defines a woman’s worth” was disputed with the idea that “my worth is self-defined.” The culturally reinforced slogan “endure for the sake of family” has, for example, been replaced with “heal for the sake of wholeness,” highlighting the change from passive endurance into active self-preservation. Similarly, the assumption that “you are alone now” after divorce was rejected in favour of “I am accompanied by myself,” emphasising emotional self-reliance.

This table integrates the content of participants’ narrative changes according to White and Epston’s (1990) narrative therapy theory, whereby people regain mastery over their stories by departing from hegemonic discourses that limit individual development. These revised scripts represent a transition away from shame and exclusion towards empowerment, dignity, and new meaning.

To enrich the analytical richness of the study and to identify converging and divergent trajectories, Table 1 provides a cross-case comparison of six participants on main narrative dimensions: family support, economic autonomy, coping strategies, type of narrative, and extent of identity reconstruction. Such a matrix for comparison enables not just thematic consistencies to be understood but also significant divergences in terms of how South Asian women cope with the consequences of divorce.

Consequently, most participants eventually reached a quest story of self-definition, empowerment, and growth; however, large differences exist in the resources and routes utilised. For instance, P1 and P3 score high on narrative reconstruction, embracing self-expression and therapy, respectively. Narrative-wise, P4 had a pathological Frank-type narrative marked by emotional stagnation and absence of identity reconstruction. This divergence serves as a reminder that empowerment is not automatic and can be constrained by intersecting vulnerabilities like low economic capital and limited family support.

Those with greater economic autonomy (e.g., P3, P5) tended to report higher agency in post-divorce identity construction, thus implying economic independence to be one of the agents facilitating narrative reframe. The families served as an alternative mechanism toward less solitude given economic hardship, i.e., greater family support (e.g., P2, P5).

The cross-case matrix points to identity reconstruction as a multidimensional process, heavily mediated by relational, cultural, and economic contextualities. These comparative results thus supplement the thematic findings toward the presentation of a highly nuanced perspective of narrative variation and ultimately affirm the value of case-based sensitivity in feminist qualitative research.

## Discussion

This research sought to comprehend the lived experience of divorce for South Asian women, a group that is habitually made invisible by dominant discourses about marriage, morality, and womanhood. Divorce for them did not present itself as a legal breakdown but as a cultural, emotional, and spiritual earthquake, overturning identity, dislocating belonging, and subjecting them to discourses of shame, failure, and abandonment. However, against this context of structural and symbolic violence, the women in this research also drew on resources of voice, body, and spirit to re-constitute their selfhood. These results not only resonate with the well-documented resilience and reinvention of marginalized women but complicate the hypothesis that life after divorce necessarily ends in empowerment. Rather, the narratives of the women disclose non-linear, embodied, and frequently contradictory processes of becoming based in the intimate landscape of emotion, memory, and ritual.

Arthur Frank’s (1995) typology of illness narrative, specifically the quest narrative offered a helpful framework by which to understand many of the study participants’ narratives. In Frank’s schema, the quest narrative transforms suffering into sense-making, allowing the narrator to find wisdom, growth, or transformation in adversity. Many of the women in this study described their post-divorce lives as quests, not just for freedom or stability but for authenticity, spiritual rootage, and emotional coherence. But close reading shows that these were not verbal or cognitive narratives alone. Instead, they were embodied: performed through yoga, stabilized through prayer, managed through breathwork, and restored through touch. This brings us to suggest a unique but analogous construct the agency quest in which the body takes center stage in the narrative of regaining life following disruption.

The agency quest diverges from the classic quest story in that it places in the forefront the materiality of healing. In the lives of these women, journaling was more than a means to record feelings—it was a physically engaged ritual of reintegration. Breathwork was not symbolic but regulative, calibrating heart rate and affect in situations of overwhelm. Yoga was not so much an expression of strength as a matter of daily boundary-taking. These practices are theoretically consonant with embodied cognition, which posits that the body is not independent of the mind but an active locus of knowing and meaning (Varela et al., 1991; Csordas, 1994). Emotional healing, in this model, is not merely accomplished by insight or narrative expression but by the body’s recursive processing of sensation, rhythm, and ritual.

Post-divorce practices among participants commonly involved intensely individualized forms of spirituality that defied patriarchal religious expectations. Most reported reconnecting with the divine, but on their own terms by rejecting shame-based dogma, calling upon feminine gods, or tuning in through daily prayer and energy work. These practices are indicative of what feminist theologians like Christ and Plaskow (1979) refer to as a “re-visioning of the sacred” where spirituality is an exercise of reclamation rather than submission. This is consonant with Mahmood’s (2005) conception of ethical self-cultivation, where religious or spiritual practice is not necessarily one of liberation from norms but of creating moral life anew from within. For the women in this research, spirituality was not about rejecting culture but about re-coding its values, language, and rituals to facilitate emotional survival.

What is striking is how often the women’s healing stories were deeply embodied and spiritual, and yet quietly revolutionary. They did not vocally repudiate cultural values but inverted them in quiet, incremental ways by embracing solitude over marriage again, self-love over sacrificial motherhood, intuition over dogmatic religion. Often, these spiritual and corporeal practices were a technology of the self (Foucault, 1988), allowing women to rewire internal scripts of shame, fear, and self-doubt. This also highlights Butler’s (2004) idea that agency is not necessarily the straightforward declaration of will, but situated performance partial, contingent, and relational. The pursuit of agency, therefore, is not about definitively triumphing over patriarchy, but about navigating its inscription through bodywork, silence, resistance, and re-authoring narrative.

But the ubiquity of these “empowerment narratives” in the data also invites critical analysis. Lacking in most of the stories were chaos stories—narratives of despair, incoherence, and lack of closure. According to Frank (1995), chaos narratives are difficult to narrate and to listen to; they resist narrative form, moral reconciliation, and the cultural appetite for redemption. It is not coincidental that such stories were lacking in this study. It could be a result of the sample (individuals who already had some level of understanding or stability), the method of data collection (e.g., podcasts, interviews, and memoirs that privilege coherent narrative), or cultural scripting against public vulnerability.

In patriarchal and collectivist societies, especially in South Asia, women are rewarded for being resilient and composed and penalized for emotional rawness and unfinished mourning. This cultural logic may lead to what Gergen and Gergen (1988) refer to as the “narrative smoothing” of life narratives where painful or uncertain experiences are edited, redefined, or kept silent in order to fit dominant norms. The structural challenge of telling about continuous suffering particularly if that suffering does not find resolution circumscribes the range of available stories for feminist knowledge-construction. Even here, when subjects expressed skepticism, terror, or hopelessness, redemptive turns, positive lessons, or spiritual revelations often followed. Such narrative turns might represent real change, but they may also represent the internalized need to give pain meaning.

As researchers, we must be acutely aware of the epistemological limits of narrative itself. What is not said is the messiness that resists resolution, the sorrow that will not abate, the relations that remain broken counts too. Herein, qualitative research risks its own “liberation bias” by strategically emphasizing testimonies of transcendence while downplaying the perpetual disarray that characterizes so many lives (Riessman, 2008; Cvetkovich, 2012). A more ethically responsive feminist praxis would vigorously make space for these fractured or ambivalent narratives, not as healing failures but as legitimate forms of post-divorce living.

Together, these accounts indicate that healing from divorce is not a linear process of empowerment but a messy, recursive, and multifaceted one. Agency among participants was not heroic but ordinary, somatic, and intimate. It meant rewriting internal scripts, negotiating cultural expectations, and rebuilding meaning through spiritual and sensory processes. In dominant public discourse, divorced women are usually constructed in binary oppositions either as victim or as victorious survivor but in their own stories, there is richer narrative ecology with detours, silences, and slow reclaimings.

The work contributes to an increasing amount of feminist and decolonial research on narrative justice (Andrews, 2020; Jackson, 2021) that highlights whose stories are being heard, by whom, how, and for what type of healing. Subsequent research should not only record increasingly diverse post-divorce experiences—those characterized by chaos, ambivalence, or loss of spirituality but must also confront the methodological and cultural forces that screen what stories can be told. Feminist praxis is particularly important in going beyond the celebration of resilience to the recognition of vulnerability and holding space for the unresolved.

## Conclusion

The research highlights the deep psychological and emotional changes that people experience after divorce. Divorce is not a relational or legal break; it is a biographical break that restructures identity, subverts embedded gender norms, and makes one redefine oneself. Through narrative research, the research opens up how South Asian women rewrite their lives by deconstructing dominant cultural scripts and aligning with more self-narratives based on autonomy, dignity, and self-love. The stories gathered corroborate that healing is not in a straight line, nor is empowerment automatic; it is contextual, relational, and highly mediated by material and affective resources.

Notably, the research adds to an increasing corpus of feminist qualitative scholarship that places the centrality of lived experience and narrative meaning-making within the purview of legitimate epistemologies. Through employing Frank’s narrative typology and expanding it via the concept of the “agency quest,” the research further illuminates how financial autonomy, social support, and spiritual activities facilitate transformational identity reconstruction. In so doing, it creates fresh theoretical space to explore how women living in collectivist and patriarchal cultures renegotiate agency within the strictures of family, culture, and institutional expectations.

Concurrently, the research team also recognises several methodological shortcomings. The purposive small sample of only three interviews and three podcast testimonies may limit thematic saturation and generalisability. As valuable as the podcast data is in terms of narrative richness, it may have added bias in favour of participants who are more articulate or tech savvy. Future studies would need to increase the sample to involve greater diversity of voices along class, caste, rural–urban locations, and sexual orientations. The addition of longitudinal methods or mixed-methods approaches could also shed light on how identity reconstruction changes over time.

This result, for the clinician, makes a case for narrative practice in post-divorce counselling. Mental health practitioners and social workers must adopt culturally competent, trauma-informed models that look at identity disruption at the core of the divorce experience. Their efforts should be underpinned by policies that invest in integrated support systems offering legal aid, mental health services, and economic empowerment programmes to address the multifaceted needs of divorced and separated women. In doing so, this makes sure stakeholders can shepherd post-divorce identity transformation as healing—in short, the divorce does not stand as an end in itself but as a socially sponsored opportunity for healing and rebirth on one’s terms.

## Implications for practice

The conclusion of this research bears serious implications as far as practice, community intervention, and gender-responsive policy are concerned. Importantly, practitioners working with divorced women, especially in South Asian contexts, should understand that post-divorce resuscitation entails both emotional healing and radical identity reconstruction. Divorces in fact wreck not only marital status but also a woman’s sense of self, social role, and existential meaning – deeply so in cultural milieus where female respectability is equated with marriage survival.

Mental health practitioners need to incorporate narrative-based therapeutic models (White and Epston, 1990) into divorce counselling. These models invite people to externalise shame, re-author disempowering narratives, and develop self-compassion. Experiences that juxtapose dominant cultural narratives (e.g., “divorce is failure”) against new self-authored narratives (e.g., “divorce is awakening”), as shown in Table 1, can assist women in rewriting their identity in empowering terms. Treaters need to be trained in trauma-informed treatment, paying heed to the sociocultural shame, silencing, and blame that South Asian women internalise.

In addition, in-person and virtual support groups must be structured not just for emotional validation but for shared meaning-making, peer mentoring, and knowledge-sharing regarding legal rights, parenting, and financial independence. Particular attention must be given to making these spaces accessible to women who have been marginalised because of their less digital empowerment and less economic empowerment in the process, whose voices get lost in dominant discourses.

The research thus underlines the imperative for policymakers to build holistic recovery ecosystems for divorced women. These ought to integrate legal aid, vocational training, trauma-informed mental health, and safe housing initiatives. As the evidence indicates, women with more robust economic means and social support were in a position to regain agency and rebuild meaning. Economic empowerment is therefore not secondary; it is central to psychological healing and social reintegration.

Lastly, practitioners and designers need to understand that healing is linear and context-specific. Divorce is not liberating for all women; for others, it makes them more entrenched in precarity and loneliness. Interventions must thus be adaptable, situated in culture, and responsive to silence, ambivalence, and resistance and not only resilience. Space needs to be made for these diverse trajectories in order to ensure that the support systems do not reproduce the same normative pressures from which women need to escape.

## Limitations and future directions

While this thesis contributes worthwhile insight, the sample size diminishes the generality of the outcome. Future research should attempt to enlarge the sample size to include a greater variation in cultural and socio-economic backgrounds. Study long-term post-divorce experiences to assess how identity reconstruction changes through time. Focus on men’s experience of divorce and compare these with women’s experience to look for gender differences. Use mixed-method approaches in gaining a deeper understanding of emotional and psychological processes.

## Data Availability

The data collected and analysed in this study are not publicly available because of the confidentiality agreement and ethical considerations. Informed consent was taken and participants were assured that their identities and responses will always remain confidential and this will not go outside the research team. Requests to access the datasets should be directed to saranya.t.sathish@gmail.com.
